# ANNOUNCEMENTS & RESOURCES

**Published:** 2019-02-10

## Obituaries

**Figure:**
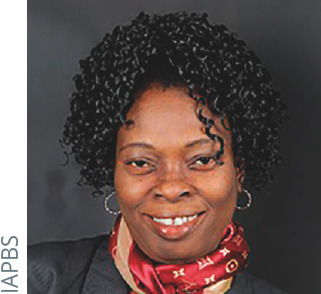


**Dr Uduak Udom** of Nigeria, the World Council of Optometry's immediate past president and a friend of the *Community Eye Health Journal*, has passed away after a long and courageous battle with illness. Read more about her remarkable career here: **http://bit.ly/UduakUdom**

**Figure:**
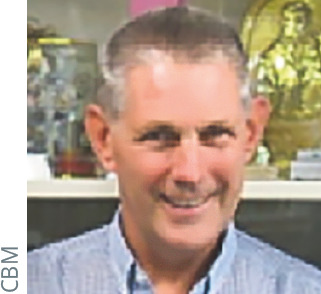


**Dr Frank Green** (UK) is remembered for his work providing ophthalmology services to refugees in Myanmar and Thailand. He performed over 20,000 cataract operations and trained selected Karen refugees to perform cataract surgery. Read more here: **http://bit.ly/DrFrankGreen**

## Useful resources

**Figure:**
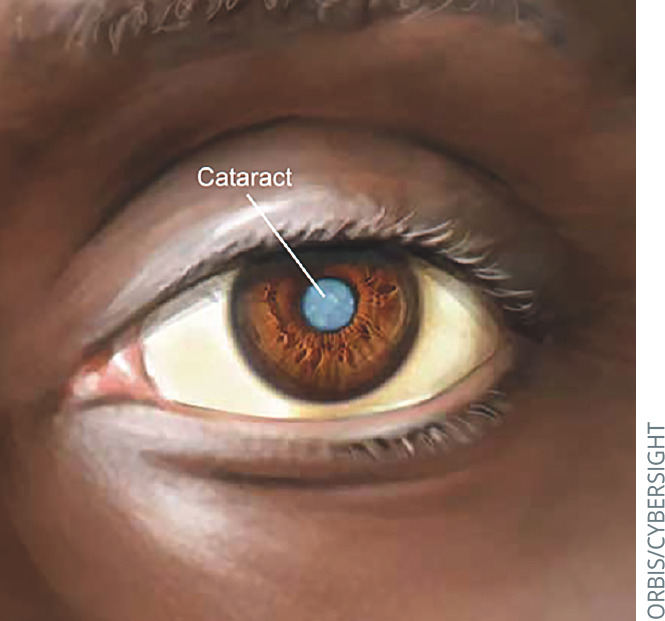


**The Eye Book: an Illustrated Guide for Patients.** Orbis has produced a book to support doctors, nurses and allied health personnel when they discuss eye diseases and conditions with patients, parents, family members and caregivers. Available for free download from Cybersight at **https://cybersight.org/portfolio/the-eye-book-an-illustrated-guide-for-patients/**

## Courses

### MSc Public Health for Eye Care, London School of Hygiene & Tropical Medicine, London, UK

Fully funded scholarships are available for Commonwealth country nationals. For more information visit **www.lshtm.ac.uk/study/masters/mscphec.html** or email **romulo.fabunan@lsthm.ac.uk**

### Small Incision Cataract Surgery Training at Lions Medical Training Centre in Nairobi, Kenya

Courses begin every six weeks and cost US $1,000 for training and approximately US $1,000 for accommodation. Email **training@lionsloresho.org** or call/message +254 728 970 601 or +254 733 619 191.

### Free online courses

**The ICEH Open Education for eye care programme** offers a series of online courses in key topics in public health eye care. All the courses are free to access. More free courses coming! Certification also available. For more information visit **http://iceh.lshtm.ac.uk/oer/**

## Subscriptions

Contact Anita Shah

**admin@cehjournal.org**

**Subscribe to our mailing list**

**web@cehjournal.org** or visit **www.cehjournal.org/subscribe**

**Visit us online**

**www.cehjournal.org**

**www.facebook.com/CEHJournal**

**https://twitter.com/CEHJournal**

